# Challenging, Safe, and Effective Use of External Iliac Vein for Insertion of Tunneled Cuffed Hemodialysis Catheters: A Single-Center Prospective Study

**DOI:** 10.1155/2022/4576781

**Published:** 2022-08-09

**Authors:** Ayman R. Abd El-Hameed, Walid A. R. Abdelhamid

**Affiliations:** Department of Internal Medicine, Zagazig University, Zagazig, Egypt

## Abstract

**Background:**

Providing well-functioning vascular access is crucial for patients undergoing chronic hemodialysis. Peripheral arteriovenous fistulas and grafts are the preferred accesses in hemodialysis patients. Patients with bilateral obstruction of internal jugular veins and subclavian veins require a suitable vascular access. Thus, the insertion of iliac vein tunneled cuffed catheters (TCCs) by interventional nephrologists may be a good option for these patients. We aimed to evaluate the outcomes of iliac vein TCCs in patients lacking other vascular options.

**Methods:**

80 tunneled cuffed hemodialysis catheters were inserted through the iliac veins of 80 patients with an end-stage kidney disease. Catheter insertion was guided by Doppler ultrasonography followed by plain radiography to detect the catheter tip and exclude complications.

**Results:**

The insertion success rate was 100%. 25 patients developed catheter-related infections. The mean survival time per catheter was 328 days. At the end of the study, 40 catheters were still functioning, 15 patients were shifted to continuous ambulatory peritoneal dialysis and 5 patients were referred to the interventional radiology department for insertion of transhepatic inferior vena cava tunneled catheters. Resistant catheter-related infection was the main cause of catheter removal in 11 patients (17.5%) in this study. Catheter malfunction was the second most common cause of catheter removal in 9 patients (11.25%).

**Conclusion:**

This study concluded that iliac vein TCCs can provide suitable vascular access in hemodialysis patients with bilateral obstruction of internal jugular veins and subclavian veins.

## 1. Introduction

Creating well-functioning vascular access is essential for the care of patients undergoing chronic hemodialysis. Peripheral arteriovenous fistulas or grafts are the access of choice because they are associated with long-term patency and a lower rate of complications according to the guidelines of the Kidney Disease Outcomes Quality Initiative (KDOQI). The use of central venous catheters for chronic hemodialysis is not recommended [1]. Central catheters have a significantly higher risk of infection and bacteremia than arteriovenous fistulas [2]. In addition, the risk of central venous stenosis or occlusion is higher than that of other access types. However, peripheral venous access is exhausted or impossible in approximately 25% of patients [3].

The recommended sites for catheter insertion are in the following order of preference according to the KDOQI guidelines: internal jugular vein, external jugular vein, femoral vein, subclavian vein, and lumbar vein [1]. Translumbar, transhepatic, and transiliac inferior vena cava tunneled cuffed catheter insertions are alternative strategies that are considered less favorable because these procedures are more difficult to perform and may be associated with a higher risk of complications [4–8].

Femoral venous catheters are prone to infection and pose an undesirable risk for thrombosis and life-threatening embolism in ambulatory patients [9]. Therefore, we evaluated the outcomes of insertion of iliac vein tunneled cuffed hemodialysis catheters (TCCs) by interventional nephrologists in patients with end-stage kidney disease (ESKD) lacking any other vascular options.

## 2. Materials and Methods

This observational prospective cohort study was carried out on a total number of 80 patients with ESKD on hemodialysis with bilateral obstruction of the internal jugular veins and subclavian veins in addition to nonfunctioning arteriovenous fistulas or grafts from April 2019 to July 2021. In this study, we successfully inserted 80 tunneled cuffed catheters using a challenging transiliac inferior vena cava approach.

The study was approved by our university hospital institutional ethics committee (ZU-IRB #6725), and written informed consent was obtained from each patient to participate in this study.

Inclusion criteria were as follows: Adult ESKD patients on regular hemodialysis with bilateral jugular and subclavian venous occlusion in addition to the failure of the fistula or graft.

Exclusion criteria were as follows: We temporarily excluded any patient with active infection or bleeding disorders until infection treatment or bleeding control.

Laboratory investigations (CBC, CRP, and coagulation profile) were done to rule out bleeding disorders and active infection. Venous system mapping using Doppler ultrasound was performed by our interventional nephrologists before catheter insertion. Doppler ultrasound evaluation included the evaluation of the internal jugular, external jugular, subclavian, femoral, and iliac veins.

We used a long-term soft tunneled catheter with a single Dacron cuff, Double-D (DD) shaped double lumens and blunt stepped tip catheters (Amecath, Egypt; *n* = 60 and Medcomp Hemo - Flow Double Lumen Catheter, USA; *n* = 20).

**Figure 1:**
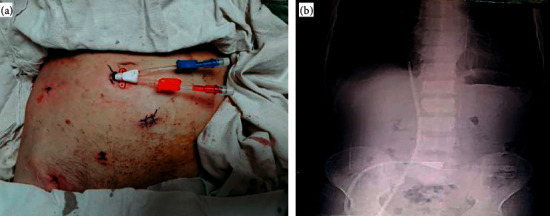
Right iliac approach of the tunneled cuffed catheter. (a) shows the position of catheter exit site below the umbilicus away from the groin making a low possibility of exit site infection. (b) Plain X-ray of the chest and abdomen shows the smooth curve of the tunnel, and the catheter tip is directed upward in the inferior vena cava till the cavo-atrial angle.

The length of the used catheter varied depending on whether it was the right or left transiliac approach. For the right iliac approach, we used the lengths of 40–55 cm depending on the patient size while for the left side, 55 cm length was used due to the long course of the left iliac vein to the right atrium (atriocaval angle). The iliac vein was approached by using the Seldinger technique [10]. The patient was placed in the supine position, and the patency of the iliac veins was assessed using Doppler ultrasound. Under sterile conditions, using a small (21 gauge) needle, 20 ml of lidocaine was administered subcutaneously, and iliac vein cannulation was performed with Doppler ultrasound guidance approximately 1 cm above the inguinal ligament at an angle of 45 degrees (Figure 1). We used at first the same anesthesia micropuncture needle as a “finder” needle to accurately identify the iliac vein and gain initial access. Next, we placed the larger introducer (18 gauge) needle immediately adjacent to the finder needle and entered the iliac vein, then the J-shaped guidewire was pushed through the introducer needle followed by the removal of the needle. A subcutaneous tunnel was created through an incision lateral to the umbilicus using a tunneler. The catheter was attached to the threaded end of the tunneler and pulled carefully through the tunnel and out via the exit site. The catheter was then introduced through a peel-away sheath. The catheter was tested using vigorous saline flushes to assess its function. The catheter exit site should be positioned near the umbilicus away from the groin and above the inguinal ligament to decrease the possibility of exit site infection [11]. The exit site was then closed, and the catheter was secured to the skin. The catheter tip is directed upward in the inferior vena cava till the cavo-atrial angle above the level of the diaphragm [12]. After catheter insertion, its position was confirmed using Doppler ultrasonography and abdominal radiography (Figure 1). Patients were followed up until the end of the study, patient death, or catheter removal.

Catheter-related infections can be categorized as catheter-related bloodstream infections (CRBSI), exit site infections, and/or tunnel infections. The presence of redness, induration, and/or tenderness ≤2 cm from the catheter exit site was indicative of an exit site infection. In the presence of discharge from the exit site, cultures were obtained before starting empirical antibiotics in the form of vancomycin. The duration of treatment was 7 to 14 days. Tenderness, hyperemia, and/or induration extending along the subcutaneous tunnel were diagnostic of tunnel infection. Cultures from drainage from the tunnel or exit site and blood cultures from the catheter lumen were obtained before the initiation of empirical antibiotics. Two blood cultures were obtained simultaneously, one from the catheter lumen and the other from a peripheral vein. Diagnosis of CRBSI depends on the presence of positive blood culture, in addition to the exclusion of other infectious sources [13].

A catheter malfunction or dysfunction was defined according to the KDOQI guidelines as failure to achieve or maintain an extracorporeal blood flow >300 ml/min at a pre-pump arterial pressure < −250 mmHg) [1]. The catheter malfunction was due to thrombus or fibrin sheath formation. The clinical diagnosis of the fibrin sheath was done clinically after simple exclusion of catheter kinking or tip malposition by doing a plain X-ray. The fibrin sheath was diagnosed if the catheter flushes easily with saline, but there is sluggish blood flow on aspiration.

Statistical analysis: The data of the participants were gathered and processed by SPSS software version 26. Means and standard deviations were used to describe continuous data while frequencies and percentages were used to describe categorical data. The iliac TCCs survival was examined using the Kaplan–Meier test. At *P* = 0.05, the difference was declared as statistically significant.

## 3. Results

The mean age of the patients was 52.8 ± 8 years and 36 (45%) patients were males. In addition to our patients in the nephrology dialysis unit of Zagazig university hospital, the patients were referred from many dialysis centers, distributed over the Sharkia governorate. The most common causes of ESKD in the study population were hypertensive nephropathy (31.25%) and diabetic nephropathy (25%) (Table 1). All patients started hemodialysis through temporary catheters and the mean catheter number was 9.41 catheters/patient. The mean duration of the well-functioning iliac TCCs was 328 days (Table 2).

Devastating early complications after insertion of iliac TCCs, such as internal bleeding, aortic perforation, or cardiac injury, did not occur. Five patients developed a post-insertion simple hematoma, while catheter-related infections were detected in 25 patients. Culture results indicated that the most common organism was *Staphylococcus aureus*, followed by *Staphylococcus epidermidis*, *E.coli*, and *Klebsiella*.

Exit site infections and tunnel infections were reported in 10 and 5 patients, respectively. Catheter malfunction or dysfunction was detected in 19 patients (23.75%). It was either due to fibrin sheath (10 events) or thrombus formation (9 events) while CRBSI was observed in 10 events: infective endocarditis (3 events) and septic shock (7 events) (Table 3).

**Figure 2:**
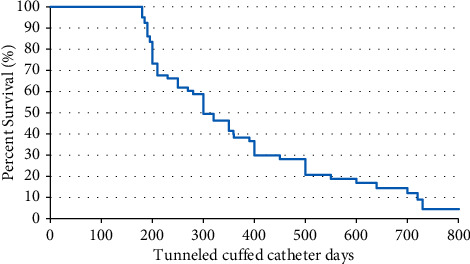
Kaplan–Meier survival curve showing survival rate of iliac vein tunneled cuffed catheter (survival probability was 75%. Mean survival was 596.267 days (95% CI: 525.671 to 668.84 days).

**Figure 3:**
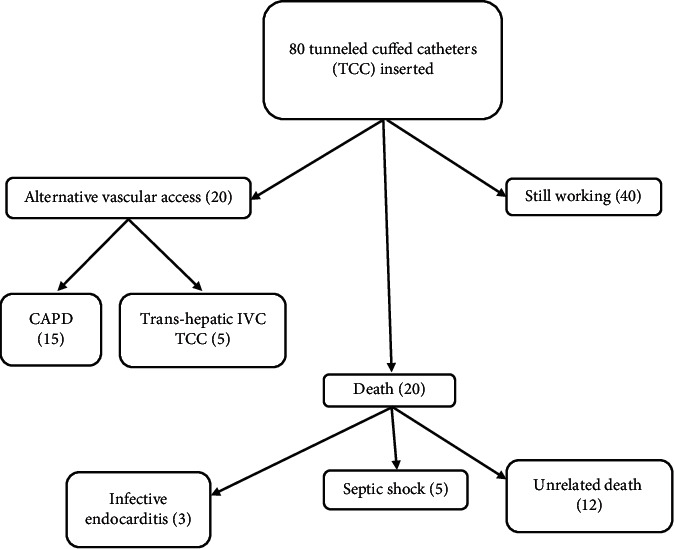
The outcome of cases at the end of the study was as follows, 40 tunneled cuffed catheters were still functioning, and 15 cases were shifted to CAPD. 5 patients were referred to the intervention radiology department for insertion of transhepatic inferior vena caval tunneled cuffed catheters. CAPD: continuous ambulatory peritoneal dialysis; IVC: inferior vena cava.

Regarding the outcomes of iliac TCCs, 50% of those hemodialysis catheters were well-functioning till the end of the study. On the contrary, resistant catheter-related infection was the main cause of catheter removal in 11 patients (17.5%) in this study. Catheter malfunction was the second cause of catheter removal in 9 patients (11.25%). The estimated survival rates of iliac TCCs were 97% at 100 days and 82% at 200 days, 69% at 400 days, and 30% at 700 days (Figure 2). Mortality unrelated to catheters was detected in 12 patients (15%), while mortality as a result of catheter-related infections occurred in 8 patients (10%) (Table 4). Finally, we summarized the outcomes of cases at the end of the study in Figure 3.

## 4. Discussion

In this research, we successfully and safely inserted a total number of 80 iliac TCCs for 80 ESKD patients with bilateral obstruction of internal jugular veins and subclavian veins. The procedure was safe and performed in our special interventional nephrology room at the nephrology dialysis unit of Zagazig university hospital without the need for vascular surgeons, interventional radiologists, or fluoroscopy assistance. So, interventional nephrologists play a very essential role in tackling the issue of difficult access to hemodialysis in hemodialysis patients by saving time and cost. The transiliac inferior vena cava approach for insertion of tunneled cuffed catheters was reported before in published studies [14, 15].

In this study, the mean duration of the well-functioning iliac TCCs was 328 days. While in previous studies of the femoral cuffed hemodialysis catheters, Restrepoy Valencia et al. [16] reported that mean survival times were 132 days in 19 cm femoral catheters and 234 days in 23 cm femoral catheters. Another study by Maya and Allon [17] showed a short femoral catheter survival time (59 days). Thus, iliac TCCs inserted from above the inguinal ligament have a longer survival time because of being away from repeated kinking by hip flexion [18].

The external iliac artery is relatively large and very close to the external iliac vein, and this may lead to arterial puncture during venous cannulation with subsequent bleeding in the form of simple hematoma up to internal bleeding [19]. The procedure was guided by ultrasound for more accurate localization of the iliac vein and to locate the catheter tip at the junction between inferior vena cava and right atrium. In our study, the bleeding was mild and controlled without clinical or radiological evidence of internal bleeding. On the other hand, previous studies on femoral catheter insertion showed a relatively higher rate of arterial puncture and hematoma as reported by Prabhu et al. [20].

The most common cause of catheter removal in our study was persistent catheter-related infection not responding to antibiotics. The frequency of infection in patients with iliac TCCs was less than that described with femoral TCCs in previous studies. Shahar et al. [21] found that the rate of CRBSI was high in patients with femoral catheters. This can be explained by the fact that the femoral catheter is located near the perineal region with a higher probability of contamination by different microbial pathogens [22, 23]. Additionally, catheter malfunction was a common cause of catheter removal in our study. This is in line with the results obtained by Wang et al. [14] and Gouda et al. [15]. In our study, catheter malfunction occurred as a result of thrombosis (11.25%) and fibrin sheath formation (12.5%), probably because of the endothelial injury of the inner layers of the iliac veins. The injury to the endothelium may be caused by catheter tip abrasion and mechanical stimulation by the catheter itself [24]. On the other hand, femoral tunneled catheters in previous studies showed lower patency rates and higher incidence rate of deep venous thrombosis as reported by Farazmand et al. [25] who found that the rate of thrombosis was 28.6% in patients with femoral tunneled catheters.

After the removal of malfunctioning or infected catheters, 15 patients were referred to peritoneal dialysis. Peritoneal dialysis was not a suitable choice from the start before transiliac tunneled catheter insertion due to a lack of family caregiving support and poor hygiene. The remaining 5 patients were referred to transhepatic inferior vena cava catheter insertion by interventional radiologists after the refusal of peritoneal dialysis. Iliac TCCs have several advantages over femoral catheters for long-term use. Iliac catheters have a lower rate of kinking with an associated lower rate of venous and/or catheter thrombosis, also infection rate is lower. Patients with iliac catheters are ambulatory without risk of catheter clotting.

## 5. Conclusion

Using the guidewire technique for placement of tunneled cuffed hemodialysis catheters as a practical method of access to the iliac vein is a safe and effective possible option for ESKD patients with difficult vascular access, especially with an increase in the number of elderly patients with multiple comorbidities.

## Figures and Tables

**Table 1:** Demographic data and patient characteristics.

Parameter	All patients (n = 80)
*Age (years), Mean* *±* *SD*	52.8 ± 8
Male sex, No (%)	36 (45%)
*Causes of ESKD*:	
(i) Hypertension, No (%)	25 (31.25%)
(ii) Diabetes mellitus, No (%)	20 (25%)
(iii) Chronic glomerulonephritis, No (%)	15 (18.75%)
(iv) Chronic pyelonephritis, No (%)	12 (15%)
(v) APCKD, No (%)	3 (3.75%)
(vi) Amyloidosis, No (%)	3 (3.75%)
(vii) Idiopathic, No (%)	2 (2.5%)
*Comorbidity*	
(i) Cerebrovascular disease, No (%)	15 (18.75%)
(ii) Congestive heart failure, No (%)	15 (18.75%)
(iii) Coronary artery disease, No (%)	25 (31.25%)
(iv) Malignancy, No (%)	5 (6.25%)
(v) Hypotension, No (%)	20 (25%)

SD: standard deviation; ESKD: end-stage kidney disease; APCKD: autosomal dominant polycystic kidney disease.

**Table 2:** Renal replacement therapy duration and vascular access history.

Parameter	All patients (*n* = 80)
Duration of RRT (years)	9.46 ± 4.25
Number of non-cuffed HD catheters	9.41 ± 2.25
AVF operations	2.3 ± 1.118
AVG operations	0.79 ± 0.65
Tunneled cuffed catheter days	328.1 ± 165.3

SD: standard deviation; RRT: renal replacement therapy; AVF: arteriovenous fistula; AVG: arteriovenous graft.

**Table 3:** Complications of catheters.

Complications of catheters	All patients (*n* = 80)
*Bleeding, No (%)*	5 (6.25%)
*Catheter thrombus formation, No (%)*	9 (11.25%)
*Fibrin sheath formation, No (%)*	10 (12.5%)
*Catheter-related infection, No (%)*	25 (31.25%)
(i) Exit site infection, No (%)	10 (12.5%)
(ii) Tunnel infection, No (%)	5 (6.25%)
(iii) Endocarditis, No (%)	3 (3.75%)
(iv) Septic shock, No (%)	7 (8.75%)

**Table 4:** Outcome of cases and endpoint of catheters.

Outcome of cases	All patients (*n* = 80)
Still working, No (%)	40 (50%)
Removal of catheter due to catheter-related infection, No (%)	11 (13.75%)
Removal of catheter due to malfunction, No (%)	9 (11.25%)
Death due to unrelated cause, No (%)	12 (15%)
Death due to catheter-related infection, No (%)	8 (10%)

## Data Availability

The data supporting the findings of this study are available from the corresponding author upon reasonable request.

## References

[1] Lok C. E., Huber T. S., Lee T. (2020). KDOQI clinical practice guideline for vascular access: 2019 update. *American Journal of Kidney Diseases*.

[2] Kumbar L., Yee J. (2019). Current concepts in hemodialysis vascular access infections. *Advances in Chronic Kidney Disease*.

[3] Krishna V. N., Eason J. B., Allon M. (2016). Central venous occlusion in the hemodialysis patient. *American Journal of Kidney Diseases*.

[4] Liu F., Bennett S., Arrigain S. (2015). Patency and complications of translumbar dialysis catheters. *Seminars in Dialysis*.

[5] Sanal B., Nas O. F., Dogan N. (2016). Safety and functionality of transhepatic hemodialysis catheters in chronic hemodialysis patients. *Diagnostic and interventional radiology*.

[6] Navuluri R., Regalado S. (2009). The KDOQI 2006 vascular access update and fistula first program synopsis. *Seminars in Interventional Radiology*.

[7] Agarwal A. K. (2013). Central vein stenosis. *American Journal of Kidney Diseases*.

[8] Polakovič V., Lopot F. (2015). Safety aspects in patients on hemodialysis with catheters. *Contributions to Nephrology*.

[9] Cheraghali R., Farshidmehr P. (2021). The patency rate of the primary and exchanged femoral haemodialysis catheters. *Malaysian Journal of Medical Sciences*.

[10] Ash A., Raio C. (2016). Seldinger technique for placement of ‘peripheral’ internal jugular line: novel approach for emergent vascular access. *Western Journal of Emergency Medicine*.

[11] Pereira K., Osiason A., Salsamendi J. (2015). Vascular access for placement of tunneled dialysis catheters for hemodialysis: a systematic approach and clinical practice algorithm. *Journal of Clinical Imaging Science*.

[12] Hill S., Moureau N. L. (2019). Tip position. *Vessel Health and Preservation: The Right Approach for Vascular Access*.

[13] Wooten R., Kothari D., Pryor R., Bearman G. (2022). Preventing hemodialysis catheter-related bloodstream infections: barriers, controversies, and best practices. *Current Infectious Disease Reports*.

[14] Wang L., Wei F., Sun G., Chen H., Yu H., Jiang A. (2016). Use of iliac vein tunneled cuffed catheters in elderly hemodialysis patients: a single-center retrospective study. *Journal of Nephrology*.

[15] Gouda Z. E., Emara M. M., Elbarbary H. S., Koura M. A. A., Elarbagy A. R. (2019). Studying alternative approaches for placement of cuffed hemodialysis catheters in hemodialysis patients with bilateral internal jugular vein occlusion. *The Journal of Vascular Access*.

[16] Restrepo Valencia C. A., Aguirre Arango J. V., Buitrago Villa C. A. (2018). Tuneled catheters in femoral vein: does the length makes any difference?. *Revista Colombiana de Nefrología*.

[17] Maya I. D., Allon M. (2005). Outcomes of tunneled femoral hemodialysis catheters: comparison with internal jugular vein catheters. *Kidney International*.

[18] Betz C., Kraus D., Müller C., Geiger H. (2012). Iliac cuffed tunnelled catheters for chronic haemodialysis vascular access. *Nephrology Dialysis Transplantation*.

[19] Copelan A., Scola D., Watts M., Ge B. (2015). Iatrogenic percutaneous vascular injuries: clinical presentation, imaging, and management. *Seminars in Interventional Radiology*.

[20] Prabhu M. V., Juneja D., Gopal P. B. (2010). Ultrasound-guided femoral dialysis access placement: a single-center randomized trial. *Clinical Journal of the American Society of Nephrology*.

[21] Shahar S., Mustafar R., Kamaruzaman L., Periyasamy P., Pau K. B., Ramli R. (2021). Catheter-related bloodstream infections and catheter colonization among haemodialysis patients: prevalence, risk factors, and outcomes. *International Journal of Nephrology*.

[22] Sahli F., Feidjel R., Laalaoui R. (2017). Hemodialysis catheter-related infection: rates, risk factors and pathogens. *Journal of Infection and Public Health*.

[23] Cheng S., Xu S., Guo J. (2018). Risk factors of central venous catheter-related bloodstream infection for continuous renal replacement therapy in kidney intensive care unit patients. *Blood Purification*.

[24] Tanabe H., Takemura N., Terao H. (2019). Vascular endothelium damage from catheter-induced mechanical stimulation causes catheter sleeve formation in a rabbit model. *The Journal of Vascular Access*.

[25] Farazmand B., Sepas H., Negahi A., Mousavie S., Vosough F. (2019). Patency and outcomes of tunneled hemodialysis catheter via femoral versus jugular vein access. *Journal of Advanced Pharmaceutical Technology & Research*.

